# The Foreign Journals: Anatomy and Physiology

**Published:** 1842-07

**Authors:** 


### PART THIRD.

### Selections from the British and Foreign Journals.

### I. THE FOREIGN JOURNALS.

### ANATOMY AND PHYSIOLOGY.

*Physiological results of Extirpation of the Salivary Glands.* By Dr. Budge.

Litmus paper, introduced into the mouths of a considerable number of rabbits, was changed into a deep blue colour. The same was the result in the case of dogs and cats, without any exception. A cat was deprived of food during two days and half, and a dog during one day; yet in both cases was the above change in the litmus paper not the less marked. A rabbit and cat had both their nervi vagi cut across: in both animals, till the moment of death, an alkaline reaction of the saliva was manifested. The cat lived till the fourth day. A dog had both its parotid, both its submaxillary, and both its sublingual glands extirpated; yet, to the astonishment of the author, the reaction was still alkaline in as great a degree as before. The glands themselves, after being washed, so as to free them from all traces of blood, were cut into and tested: the reaction was alkaline, but not in so great a degree as when the paper was introduced into the mouth. The animal quite recovered, and during the four weeks which it was permitted to survive, litmus paper introduced into the mouth, was always tinged blue. It was killed, and on examination a small quantity of food was found in its stomach. One bit of litmus paper laid on the stomach remained unaltered in colour; another piece became slightly reddened. The acid reaction seemed less than usual; but the author adds, that in dogs whose glands had not been extirpated he has often noticed very faint traces of acid. The results in a cat, which survived the operation four weeks without the smallest apparent injury, were nearly identical.

From these experiments the author infers that the spleen is not the only gland which can be extirpated without destruction of life. Yet no one can suppose that the salivary glands are useless. We must therefore conjecture that certain glands supplement each other; and that in the case of the removal of the salivary glands the pancreas, perhaps, eliminates the fluid which these glands usually do. It is known for certain that even the urinary secretion cannot remain in the blood when the kidneys are extirpated, and that the stomach endeavours in this case to eliminate, and *does* eliminate, genuine urine, though not, of course, in quantity sufficient for the purposes of life.

Although, according to experiments and observations on the human subject, the saliva, after eating, manifests an alkaline reaction, yet section of the vagus in brutes produces no change. The sympathy between parts supplied by the vagus and those supplied by the trigemini cannot here be taken into consideration. It is possible that this sympathy between the stomach and salivary glands may take place through the spinal branches received by the parotid.

*Medicinische Zeitung*, No. xviii. *Mai* 4, 1842.

*On the Nerves of the Hard Palate.* By Professor BOCHDALEK, of Prague.

As soon as the branches of the posterior palatine nerve have passed through their foramen they separate. Three or four small branches go backwards and inwards to the glands of the soft palate; others somewhat larger pass outwards to the gum by the last teeth; and the largest fasciculus, composed of five or six branches, goes forwards, gradually diverging and forming a kind of network over the whole arch of the palate. Its outer margin lies in the gums of all the teeth, and the inner forms the most varied plexuses at the middle line, with corresponding branches from the nerve of the other side. The number of filaments into which these five or six branches divide is indeed surprising. They lie even in several layers one over another, and form close networks, so that the sum of the branches seems to surpass by very far that of the trunks. Some of their filaments pass between the teeth to the anterior part of the gum, and there unite with the terminal branches of the supra-maxillary nerve which penetrate the anterior part of the alveolar process. But innumerable branches are lost in the glands of the hard palate and in their acini.

The author suggests that the purposes served by these nerves are, to give the energy necessary to so much reproduction as is constantly going on in the hard palate, to give sensibility to the mucous membrane of the lower part of the nasal cavities, and perhaps to add filaments to those of the superior maxillary nerve in the substance of the jaw. But higher uses he thinks they have in rendering the hard palate a delicate organ of touch: and he urges it as evidence for Panizza's view of the glosso-pharyngeal nerve being the exclusive nerve of taste, that its filaments are sent to the soft palate in which there is a distinct sense of taste; while the hard palate, which has no such sensibility, is supplied by the fifth nerve alone.

*Medicinische Jahrbücher des K. K. Oesterr. States. Jan.* 1842.

---

*On the Anterior Columns of the Spinal Cord.* By Dr. STILLING, of Cassel.

IN frogs, the division of the white substance of the anterior part of the spinal cord, if the gray central substance be not injured, does not prevent the influence of the will upon the muscles which are supplied with nerves from below the wounded part. For a time the frog remains dull and as if asphyxiated; but he soon begins to move, and at length succeeds in obtaining sufficient power over his hind legs to leap for some distance with them.

Van Deen had already shown that sensitive impressions are conveyed through the posterior gray matter; and these seem to prove that the influence of the will is conveyed through the anterior part of the same substance; for if the incision be carried through the latter, all power of voluntary motion is lost.

*Oppenheim's Zeitschrift für die gesammte Medicin. Januar,* 1842.

---

*On the Fatty Substance of Milk.* By M. DE ROMANE.

THE author, in a memoir read at the Institute on the 25th of April, asserts, that in the formation of butter by churning there is no chemical change in the fatty constituent of milk; but that by the constant agitation of the fine membranous cells in which the particles of the butter, composing the milk-globules, are contained are broken, and the particles themselves are thus allowed to come into contact, and adhere in a uniform mass. Butter-milk, he adds, owes its opalescent appearance to the particles of the envelopes of the globules, which are suspended in it in vast numbers. He believes that this construction of the globules is adapted to the nutrition of the young animal; the membranes serving to shield the particles of butter till they have arrived in the stomach.

*Gazette Médicale. Avril* 30, 1842.

*Closure of the Nasal Fossæ without "Nasillement."*

THERE is at present under M. Lisfranc a man, in whom, in consequence of injury producing necrosis of some of the bones of the face, the whole of the soft palate is firmly adherent to the back of the pharynx, so that there is on this side no posterior aperture of the nares. Yet he does not "speak through his nose." And when the aperture which remains from the pharynx into the nose is carefully closed with a plug, his voice still remains unaffected.

*Bulletin Gén. de Thérapeutique. Mars,* 1842.

---

*On the Origin, Mode of Formation, and End of the Blood Globules.*
By M. DONNE.

IN this memoir the author maintains that the white globules of the blood are formed by the aggregation of chyle-globules, and are intermediate in development between the latter and the true blood-globules. He adds two novelties: 1st, that milk-globules injected into the blood undergo the same changes as those of the chyle, and are like them converted into blood-globules; and 2d, that the blood-globules, after a certain period of existence, are dissolved into the liquor sanguinis.

*Gazette Médicale. Mars* 12, 1842.

---

*On the Cause of the first Respiration after Birth.* By DR. JULIUS BUDGE, of Bonn.

THE experiments detailed in this paper hardly merit notice except for the purpose of expressing a thorough disgust at their brutality and uselessness. It has been long known that division of the vagi nerves renders the respiration slower: and it is concluded that through them the impressions are conveyed from the lungs to the medulla oblongata, which, being reflected, excite the respiratory movements. But Dr. M. Hall has made it probable that the first respiration is due to a reflection of the impression produced by the cold air upon the skin of the new-born animal; and it has been suggested that after birth also the skin is one of the recipients of the centripetal impressions on which respiration depends. To prove this Dr. Budge actually removed daily a considerable portion of the skin from a rabbit, two crows, and several frogs. The experiments on the frogs were all useless; nothing, he admits, could be deduced from them. In the crows and the rabbit—who survived till, by several instalments, they were almost completely flayed—the respiration was generally, but not always, slower than is natural; the flaying seemed to have an effect somewhat like that of dividing the vagi nerves.

And now, who thinks it more probable than it was before, that impressions on the skin excite the movements of respiration? Who but he would forget that the sensitive nervous filaments exist as well in the raw skin which his cruelty exposes, as in that which he removes?

The details of the experiments are to be found in

*Casper's Wochenschrift. Marz 19th, 26th,* 1842.

---

*On Apoplexia Intermeningea.* By DR. JOSEPH ENGEL.

THE author remarks, that the sanguineous extravasation in this case, although it becomes in the course of time a firm, membrane-like substance, of a rusty hue, never presents the slightest organization, in which it differs from the true exudation, although no proper physiological explanation of this difference has been given.

*Oesterr. Medicinische Wochenschrift,* No. ix. *Feb.* 26, 1842.

